# Soil-Transmitted Helminths in Southwestern China: A Cross-Sectional Study of Links to Cognitive Ability, Nutrition, and School Performance among Children

**DOI:** 10.1371/journal.pntd.0003877

**Published:** 2015-06-25

**Authors:** Chengfang Liu, Renfu Luo, Hongmei Yi, Linxiu Zhang, Shaoping Li, Yunli Bai, Alexis Medina, Scott Rozelle, Scott Smith, Guofei Wang, Jujun Wang

**Affiliations:** 1 Center for Chinese Agricultural Policy, Institute of Geographical Sciences and Natural Resource Research, Chinese Academy of Sciences, Beijing, China; 2 University of Chinese Academy of Sciences, Beijing, China; 3 Freeman Spogli Institute, Stanford University, Stanford, California, United States of America; 4 Stanford University School of Medicine, Stanford, California, United States of America; 5 National Institute of Parasitic Diseases, Chinese Center for Disease Control and Prevention, Shanghai, China; Australian National University, AUSTRALIA

## Abstract

**Background:**

Empirical evidence suggests that the prevalence of soil-transmitted helminth (STH) infections in remote and poor rural areas is still high among children, the most vulnerable to infection. There is concern that STH infections may detrimentally affect children’s healthy development, including their cognitive ability, nutritional status, and school performance. Medical studies have not yet identified the exact nature of the impact STH infections have on children. The objective of this study is to examine the relationship between STH infections and developmental outcomes among a primary school-aged population in rural China.

**Methodology/Principal Findings:**

We conducted a large-scale survey in Guizhou province in southwest China in May 2013. A total of 2,179 children aged 9-11 years living in seven nationally-designated poverty counties in rural China served as our study sample. Overall, 42 percent of the sample’s elementary school-aged children were infected with one or more of the three types of STH—*Ascaris lumbricoides* (ascaris), *Trichuris trichuria* (whipworm) and the hookworms *Ancylostoma duodenale* or *Necator americanus*. After controlling for socioeconomic status, we observed that infection with one or more STHs is associated with worse cognitive ability, worse nutritional status, and worse school performance than no infection. This study also presents evidence that children with *Trichuris* infection, either infection with *Trichuris* only or co-infected with *Trichuris* and *Ascaris*, experience worse cognitive, nutritional and schooling outcomes than their uninfected peers or children infected with only *Ascaris*.

**Conclusions/Significance:**

We find that STH infection still poses a significant health challenge among children living in poor, rural, ethnic areas of southwest China. Given the important linkages we find between STH infection and a number of important child health and educational outcomes, we believe that our results will contribute positively to the debate surrounding the recent Cochrane report.

## Author Summary

Empirical evidence suggests that the prevalence of soil-transmitted helminth (STH) infections in remote and poor rural areas is still high among children, the most vulnerable to infection. There is concern that STH infections may detrimentally affect children’s healthy development, including their cognitive ability, nutritional status, and school performance. Medical studies have not yet identified the exact nature of the impact STH infections have on children. The objective of this study is to examine the relationship between STH infections and developmental outcomes among a primary school-aged population in rural China. We conducted a large-scale survey in Guizhou province in southwest China in May, 2013. Overall, 42 percent of elementary school-aged children were infected with one or more of the three types of STH—*Ascaris lumbricoides* (ascaris), *Trichuris trichuria* (whipworm) and the hookworms *Ancylostoma duodenale* or *Necator americanus*. After controlling for socioeconomic status, we observed that children infected with one or more STHs have worse cognitive ability, worse nutritional status, and worse school performance than their uninfected peers. While not causal, this study also presents evidence that children with *Trichuris* infection, either infection with *Trichuris* only or co-infected with *Trichuris* and *Ascaris*, have worse cognitive, nutritional and schooling outcomes than their uninfected peers or children infected with only *Ascaris*. Given these important linkages, we hope that our results will contribute positively to the debate surrounding the recent Cochrane report.

## Introduction

A recent Cochrane Report [1] has raised questions about the nature of the relationship between infection with soil-transmitted helminths (STHs) and children’s healthy development. In a meta-analysis of 42 papers, the authors of the report found that there was no clear, consistent relationship between deworming and improvements in children’s cognitive ability, nutritional indicators, or school performance. The report ended with a call for more concerted research that would help clarify the nature of the relationship between STH infection and these outcomes in children. Health policymakers depend on this type of information when deciding how to allocate resources to different disease types, in general, and how to allocate for STH control and treatment, in particular.

Because of high STH prevalence, China is an especially suitable setting in which to conduct such additional research. According to Wang et al. [2], 40 percent of school-aged children in rural areas of Guizhou province are infected with one or more types of three STHs: *Ascaris lumbricoides* (ascaris), *Ancylostoma duodenale* (hookworm), and *Trichuris trichuria* (whipworm). In some villages, the prevalence is as high as 80 percent. Similarly high prevalence has also been reported elsewhere in China, such as in rural Guangxi and Hainan provinces [3].

The uncertainty in the international literature about the link between STH infections and child outcomes is reflected in the China-specific literature. Of six total China-based studies, five measured the link between STH infection and child health (either physical development or hemoglobin levels). Three of these [2–4] found a significant negative correlation between STH infection and children’s health, while two found no correlation [5–6]. None of the six studies measured school performance, although three attempted to measure the relationship between STH infections and cognitive ability using formal tests; however, the sample sizes in these studies were small, ranging from 140 to 200 children in 2 to 11 communities or clusters. With such small sample sizes, it is statistically improbable to produce meaningful results. In short, in the context of China—as in the rest of the world—there is uncertainty about the relationship between STH infections and child outcomes.

In this paper we will answer questions raised by the Cochrane report [1] and build evidence on the relationship between STH infections and health outcomes in children in rural China. To achieve this goal, we have three objectives. First, we will document the prevalence of STHs in the study areas, thus better defining the severity of the STH problem in poor areas of rural China. Second, we will document the levels of cognitive ability, nutritional indicators and school performance among our sample children in order to assess how children in poor rural areas fare in terms of these measured outcomes. Finally, we will examine the links between STH infection and cognitive ability, nutritional indicators, and school performance.

## Methods

### Sample Selection

**Fig 1:**
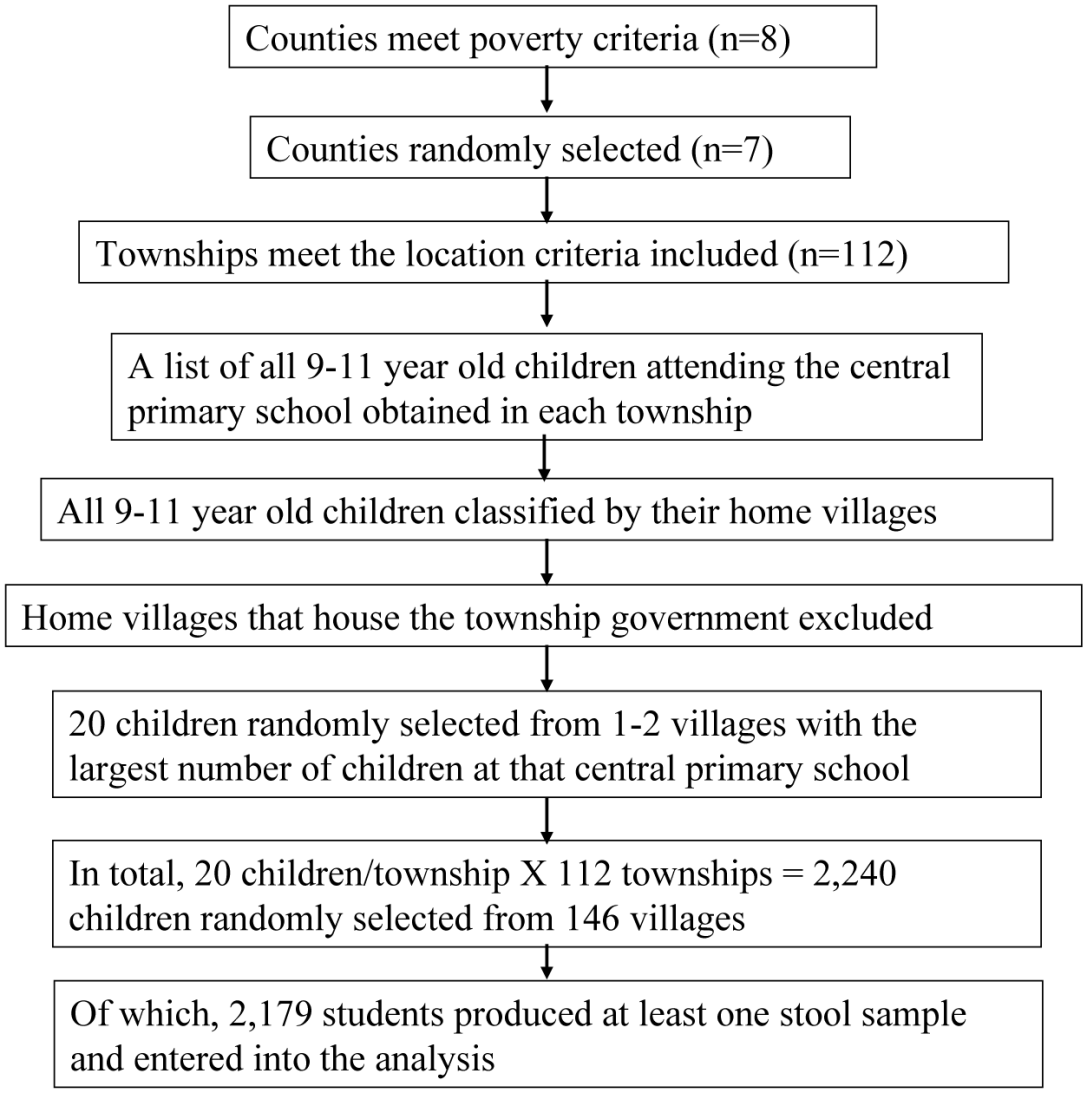
Sample selection process.

We collected the data used in May of 2013 as part of a large-scale survey of elementary school-aged children in Guizhou province. Our study was conducted in seven rural counties in Qiandongnan prefecture. We chose our sample to include regions that were poor and populated by ethnic minorities, the subpopulations that are at higher risk for STH infection. Fig 1 depicts the sample selection process. Based on rural per capita income levels reported in Guizhou Statistical Yearbook [7], the research team randomly selected a total of seven rural counties from the poorest half of the counties (8 out of 16) in Qiangdongnan. According to national statistics, at 4,625 yuan, the average rural individual in our sample areas has a per capita income in the bottom quartile of China’s rural income distribution [8].

Once we chose the sample counties, we selected the sample townships and villages. In each county, we included all townships except for the township which houses the county government. We did not include the township that housed the county government because such townships are almost always wealthier and more urban than a typical rural township. A total of 112 townships were selected. Then sample villages within each township were selected. Since our survey would take place in schools, we obtained a list of all the 9–11 year old children attending the central primary school in each township. We classified all 9–11 year old children by their home village, and then randomly selected 20 sample children from the home village with the largest number of children at that school. We excluded villages that housed the local township government, since (as discussed above in the context of towns/counties) these villages are typically wealthier and more urban than a typical village. If the home village had fewer than 20 children in our age group attending the school, we randomly selected children from the next-largest village to fill in the gap. Overall, a total of 20 school children were randomly chosen from either one or two villages in each township. A total of 2,240 children from 146 villages and 112 townships in the 7 poor rural counties were chosen as sample students.

### Data Collection

The survey team collected four types of information: data from a socioeconomic survey; scores on a test of cognitive ability; measures of child health (including STH infection status, height, weight, and hemoglobin levels); and school absence and performance (as measured by absenteeism and performance on a standardized math test).

#### Socioeconomic survey

The socioeconomic survey collected data on children and parents’ basic demographic information, as well as data on each child’s self-reported health, home and school sanitation behavior, and a series of basic questions about household conditions. The survey also asked both whether the child had taken anti-helminth medication in the past 6 months and whether they had taken it in the past 12 months. The school children completed the survey themselves under the direct monitoring of trained enumerators from the Chinese Academy of Sciences and Guizhou University of Finances and Economics.

#### Cognitive ability

Cognitive ability was assessed using a battery of four sub-tests taken from the Mandarin-language version of the Wechsler Intelligence Scale for Children Fourth Edition (WISC-IV) (See Table A in S1 Text). As the latest version, WISC-IV was culturally adapted, translated and edited into simplified Chinese and validated for Chinese children based in 2008 [9]. Since research suggests that children’s working memory and processing speed are those areas of cognitive ability most likely to be affected by STH infection [3, 10–14], we focused our efforts on measuring these two outcomes. In WISC-IV, the working memory index (WMI) is assessed through two core subtests: Digit Span, and Letter Number Sequencing. The Processing Speed Index (PSI) is assessed through two other core subtests: Coding, and Symbol Search. Raw scores obtained from these core subtests were converted to age-scaled index scores using tables of norms from the official WISC-IV administration and scoring manual for China.

Each of the 2,240 sample children was individually administered the four core sub-tests by trained examiners.

#### Health indicators

We focus on three health indicators: hemoglobin concentrations (Hb), height and weight. Hemoglobin levels were measured on-site using HemoCue Hb 201+ systems. Height and weight measurements were also taken on site, following WHO standard protocol [15]. The children were measured in light clothing without shoes, hats or accessories. Weight was measured with a calibrated electronic scale recommended by scholars from the West China School of Public Health of Sichuan University. Body height was measured using a standard tape measure. The nursing team was trained to ensure that the weighing station was set up on level ground to ensure accuracy of the equipment. Two nurses manned each measurement station, with one responsible for preparing subjects for measurement (removing shoes, offering instruction, reassuring parents, positioning children, etc.) and the other responsible for conducting and recording the measurements.

#### School absence and performance

School absence was measured by school absenteeism based on reports by each student’s homeroom teacher in official school records. To analyze the absenteeism data, we created a dummy variable that is equal to one if a student had ever been absent since the beginning of the semester, and zero otherwise. School performance was measured by a standardized math test administered by the study team (Trends in International Mathematics and Science Study, or TIMSS). The TIMISS test has been widely used in the context of poor rural areas of China to measure the school performance of children at relevant grades [16–19].

#### Stool sample collection and testing

The study team collected two stool samples from each child in our sample, one stool sample per day for two consecutive days. Samples were picked up once per day by the study team, and were stored in a temperature-controlled cooler until collection. At the time of collection, members of the study team transported all stool samples in a temperature-controlled cooler to the laboratory of the local Center for Disease Control (CDC) located at the county seat. All stool samples were tested the same day on which they were collected. A total of 2,179 children produced at least one stool sample, and 75 percent of children produced two stool samples.

All stool samples were analyzed using the Kato-Katz smear method for *Ascaris lumbricoides* (Ascaris), *Trichuris trichuria* (whipworm), and *Ancylostoma duodenale* or *Necator americanus* (hookworm). Two smears were taken from each of the two stool samples collected from each child: one smear was tested the same day on-site (for a total of two per child). The remaining smear from each sample was treated using a formaldehyde preservation technique and sent to the headquarters of the National Institute for Parasitic Diseases in Shanghai for a quality check. Children were considered positive for STH infection if either one of their stool samples tested positive for one or more types of STH.

### Ethics Statement

This study received ethical approval from the Stanford University Institutional Review Board (IRB) (Protocol ID 25027), and from the Sichuan University Ethical Review Board (Protocol ID 2013005–02). All participating children gave their assent for their involvement in the study, and the children’s legal guardians gave their written consent for both their own and their children’s involvement. Children who were found to have severe anemia were referred to the local hospital for treatment.

### Statistical Analysis

Anemia status was determined based on finger prick blood analysis for hemoglobin (Hb). Following internationally accepted standards, anemia was defined as Hb<115 g/L [20].

Physical indicators of height and weight were used to construct height-for-age z-scores (HAZ) and Body Mass Index (BMI)-for-age z-scores using WHO AnthroPlus, a software application of the WHO Reference 2007 for children aged 5–19 years that is used to monitor the growth of school-aged children and adolescents [21]. Weight-for-age z-scores (WAZ) were calculated using a SAS program for the 2000 CDC growth chart for children aged 0–20 years [22]. We followed internationally recognized cutoffs [23] to consider children whose HAZ, WAZ, or BMI-for-age z-score to fall more than two standard deviations below the international mean to be stunted, underweight, or malnourished, respectively.

Raw scores obtained from the four core subtests of the WISC-IV were converted to age-scaled index scores using tables of norms in the Mandarin version of the WISC-IV administration and scoring manual. Two index scores are considered for analysis: Working Memory Index (WMI) and Processing Speed Index (PSI). Scores are divided into internationally-recognized ranges. A score of 90–110 is considered “average”; a score of 80–89 is considered “low average”; a score of 70–79 is considered “borderline”; and a score of below 70 is considered “extremely low” and at risk for intellectual disabilities or mental retardation.

All statistical analyses were performed using STATA 12.0. P-values below 0.05 were considered statistically significant. All P-values were adjusted for multiple hypotheses testing by the Bonferroni method. The statistical significance of differences in all outcomes by subgroup populations was assessed using student’s t-test in STATA. STATA’s multiple linear regression model was used in the multivariate analysis for those continuous outcome variables: WMI, PSI, Hb, HAZ, WAZ, BmiAZ, as well as standardized math test scores. Meanwhile, STATA’s logistic regression model was used in the multivariate analysis for those binary outcome variables: anemic (yes/no) and school absence (yes/no). We included the following independent variables as potential confounders in the multivariate analysis: gender, age, boarding status, minority status, sanitation behaviors, and household characteristics. Definitions of key variables to be used in the rest of the paper are presented in Table B in S1 Text.

## Results

We examined 2,179 school-aged children. In our sample, 46 percent of the students were female and 54 percent were male, a ratio similar to those found in most poor areas in China [24]. The average age is 10.6 years. A total of 26 percent of sample students board at school.

The background characteristics of the sample by infection status are presented in Table 1. There are no significant differences between the infected and uninfected group in terms of deworming history or gender. However, infected children are more likely to be older, to board at school, and to be a member of the Miao, or Shui ethnic minority groups. Infected children are also significantly more likely than uninfected children to eat uncooked meat, drink unboiled water, and to live in households with a dirt floor. Uninfected children are significantly more likely than infected children to wash their hands after using the toilet, to live in households with a private latrine, to have a higher household income, and to have better educated parents.

**Table 1:** Background characteristics by STH infection status, values are mean (SD) [95% Confidence Interval].

Variable	Full sample (n = 2179)		Children Uninfected (n = 1267)		Children Infected (n = 912)		t-test H0: (2) = (3)
	(1)		(2)		(3)		(4)
***Individual characteristics***							
(1) Dewormed in past 6 months	0.12 (0.32)	[0.10, 0.13]	0.12 (0.32)	[0.09, 0.13]	0.12 (0.32)	[0.11, 0.15]	0.95
(2) Dewormed in past 12 months	0.18 (0.38)	[0.16, 0.19]	0.18 (0.38)	[0.14, 0.19]	0.17 (0.38)	[0.16, 0.21]	0.58
(3) Female	0.46 (0.50)	[0.44, 0.48]	0.48 (0.5)	[0.46, 0.52]	0.44 (0.5)	[0.40, 0.46]	0.14
(4) Age	10.58 (0.87)	[10.55, 10.62]	10.54 (0.87)	[10.50, 10.61]	10.65 (0.85)	[10.56, 10.66]	<0.01
(5) Boarder	0.26 (0.44)	[0.24, 0.28]	0.24 (0.42)	[0.22, 0.27]	0.30 (0.46)	[0.25, 0.30]	<0.01
(6) Minority	0.90 (0.30)	[0.89, 0.91]	0.90 (0.30)	[0.88, 0.92]	0.90 (0.29)	[0.89, 0.92]	0.75
(7) --Dong	0.45 (0.50)	[0.43, 0.47]	0.51 (0.50)	[0.41, 0.46]	0.37 (0.48)	[0.44, 0.50]	<0.01
(8) --Miao	0.37 (0.48)	[0.35, 0.39]	0.32 (0.47)	[0.35, 0.41]	0.43 (0.5)	[0.33, 0.39]	<0.01
(9) --Shui	0.04 (0.19)	[0.03, 0.04]	0.02 (0.15)	[0.03, 0.06]	0.06 (0.23)	[0.02, 0.04]	<0.01
(10) --Zhuang	0.02 (0.13)	[0.01, 0.02]	0.02 (0.14)	[0.02, 0.04]	0.01 (0.12)	[0.00, 0.01]	0.34
(11) --Other minority	0.03 (0.16)	[0.02, 0.03]	0.02 (0.15)	[0.01, 0.03]	0.03 (0.17)	[0.02, 0.05]	0.39
***Eating and sanitation***							
(12) Wash hands before eating	0.84 (0.36)	[0.83, 0.86]	0.85 (0.36)	[0.82, 0.86]	0.84 (0.37)	[0.82, 0.87]	0.44
(13) Wash hands after using toilet	0.87 (0.34)	[0.85, 0.88]	0.89 (0.32)	[0.84, 0.88]	0.84 (0.36)	[0.86, 0.90]	<0.01
(14) Never eats uncooked vegetables	0.31 (0.46)	[0.29, 0.33]	0.31 (0.46)	[0.28, 0.33]	0.31 (0.46)	[0.29, 0.34]	0.75
(15) Never eats uncooked meat	0.62 (0.48)	[0.60, 0.64]	0.68 (0.47)	[0.59, 0.65]	0.54 (0.50)	[0.60, 0.66]	<0.01
(16) Never drinks un-boiled water	0.07 (0.25)	[0.06, 0.08]	0.06 (0.24)	[0.07, 0.10]	0.08 (0.27)	[0.04, 0.07]	0.06
(17) Never being outside with bare feet	0.33 (0.47)	[0.31, 0.35]	0.33 (0.47)	[0.30, 0.36]	0.33 (0.47)	[0.30, 0.35]	0.95
(18) Dirt floor	0.16 (0.36)	[0.14, 0.17]	0.13 (0.33)	[0.12, 0.16]	0.20 (0.40)	[0.15, 0.19]	<0.01
(19) Own toilet	0.85 (0.36)	[0.83, 0.86]	0.89 (0.32)	[0.82, 0.87]	0.79 (0.40)	[0.83, 0.87]	<0.01
(20) Dirt-based latrine	0.21 (0.41)	[0.20, 0.23]	0.19 (0.39)	[0.21, 0.26]	0.25 (0.43)	[0.17, 0.22]	<0.01
(21) Uses night soil	0.64 (0.48)	[0.62, 0.66]	0.67 (0.47)	[0.59, 0.65]	0.60 (0.49)	[0.63, 0.68]	<0.01
***Household characteristics***							
(22) Household size	5.27 (1.41)	[5.21, 5.33]	5.34 (1.41)	[5.23, 5.40]	5.18 (1.39)	[5.15, 5.32]	0.01
(23) Siblings	1.18 (0.94)	[1.14, 1.22]	1.16 (0.92)	[1.18, 1.29]	1.21 (0.97)	[1.07, 1.18]	0.30
(24) Pieces of durable assets	8.38 (3.13)	[8.24, 8.51]	8.83 (3.12)	[8.11, 8.50]	7.74 (3.03)	[8.27, 8.63]	<0.01
(25) Neither parent present	0.30 (0.46)	[0.28, 0.32]	0.34 (0.47)	[0.26, 0.31]	0.25 (0.43)	[0.29, 0.34]	<0.01
(26) Mother secondary school	0.07 (0.25)	[0.06, 0.08]	0.08 (0.28)	[0.05, 0.08]	0.05 (0.22)	[0.06, 0.09]	<0.01
(27) Father secondary school	0.12 (0.32)	[0.10, 0.13]	0.13 (0.34)	[0.09, 0.13]	0.09 (0.29)	[0.10, 0.14]	<0.01

Source: authors’ survey.

### STH Prevalence

Of the 2,179 children who provided fecal samples, participated in the socioeconomic survey, cognitive testing, health examination as well as school absence and performance tests, 42 percent were infected with one or more of the three types of STH (Table 2). The most prevalent type of STH in the survey areas is *Ascaris* (31 percent), followed by *Trichuris* (22 percent), and finally by hookworm (1 percent). The prevalence of *Ascaris* only and *Trichuris* only was 19 percent and 11 percent, respectively. Eleven percent of sample children were co-infected with *Ascaris* and *Trichuris*. In contrast, the prevalences of infection with hookworm only, co-infection with *Ascaris* and hookworm, co-infection with *Trichuris* and hookworm, or co-infection with *Ascaris*, *Trichuris* and hookworm were negligible.

**Table 2:** Cognitive ability, nutritional indicators, school absence and performance, by STH infection status. Values are mean (SD) [95% Confidence Interval].

Variable	Full sample (n = 2179)		Children Uninfected (n = 1267)		Children Infected (n = 912)		P-Value H0: (2) = (3)
	(1)		(2)		(3)		(4)
***STH infection***							
(1) Any STH infection	0.42 (0.49)	[0.40, 0.44]	0.00 (0.00)	[0.00, 0.00]	1.00 (0.00)	[1.00, 1.00]	na
(2) Ascaris	0.31 (0.46)	[0.29,0.33]	0.00 (0.00)	[0.00, 0.00]	0.73 (0.44)	[0.70, 0.76]	na
(3) Trichuris	0.22 (0.42)	[0.21,0.24]	0.00 (0.00)	[0.00, 0.00]	0.54 (0.50)	[0.50, 0.57]	na
(4) Hookworm	0.01 (0.07)	[0.00,0.01]	0.00 (0.00)	[0.00, 0.00]	0.01 (0.11)	[0.00, 0.02]	na
(5) Ascaris only	0.19 (0.39)	[0.17, 0.21]	0.00 (0.00)	[0.00, 0.00]	0.46 (0.50)	[0.42, 0.49]	na
(6) Trichuris only	0.11 (0.31)	[0.10, 0.12]	0.00 (0.00)	[0.00, 0.00]	0.27 (0.44)	[0.24, 0.29]	na
(7) Hookworm only	0.00 (0.04)	[0.00, 0.00]	0.00 (0.00)	[0.00, 0.00]	0.00 (0.07)	[0,00. 010]	na
(8) Ascaris + Trichuris	0.11 (0.31)	[0.10, 0.12]	0.00 (0.00)	[0.00, 0.00]	0.27 (0.44)	[0.24, 0.29]	na
(9) Ascaris + Hookworm	0.00 (0.04)	[0.00, 0.00]	0.00 (0.00)	[0.00, 0.00]	0.00 (0.06)	[0.00, 0.01]	na
(10) Trichuris + Hookworm	0.00 (0.02)	[0.00, 0.00]	0.00 (0.00)	[0.00, 0.00]	0.00 (0.03)	[0.00, 0.00]	na
(11) Ascaris + Trichuris + Hookworm	0.00 (0.04)	[0.00, 0.00]	0.00 (0.00)	[0.00, 0.00]	0.00 (0.06)	[0.00, 0.01]	na
***Cognitive ability***							
(12) Working memory index (WMI)	78.60 (9.93)	[78.18, 79.01]	80.02 (10.41)	[79.45, 80.59]	76.62 (8.87)	[76.04, 77.19]	<0.01
(13) --Extremely low WMI (<70)	0.13 (0.34)	[0.12, 0.15]	0.11 (0.31)	[0.09, 0.13]	0.16 (0.37)	[0.14, 0.19]	<0.01
(14) --Borderline WMI (70–79)	0.50 (0.50)	[0.48, 0.52]	0.46 (0.50)	[0.43, 0.49]	0.55 (0.50)	[0.52, 0.58]	<0.01
(15) --Extremely low / borderline WMI (<80)	0.63 (0.48)	[0.61, 0.65]	0.57 (0.50)	[0.54, 0.60]	0.71 (0.45)	[0.68, 0.74]	<0.01
(16) Processing speed index (PSI)	86.15 (13.11)	[85.60, 86.71]	88.14 (12.76)	[87.44, 88.85]	83.39 (13.09)	[82.54, 84.24]	<0.01
(17) --Extremely low PSI (<70)	0.10 (0.30)	[0.09, 0.11]	0.07 (0.26)	[0.06, 0.09]	0.14 (0.35)	[0.12, 0.17]	<0.01
(18) --Borderline PSI (70–79)	0.26 (0.44)	[0.24, 0.27]	0.22 (0.42)	[0.20, 0.24]	0.30 (0.46)	[0.27, 0.33]	<0.01
(19) --Extremely low / borderline PSI (<80)	0.36 (0.48)	[0.34, 0.38]	0.29 (0.46)	[0.27, 0.32]	0.45 (0.50)	[0.41, 0.48]	<0.01
***Nutritional indicators***							
(20) Hb	126.25 (12.45)	[125.73, 126.77]	126. 84(11.95)	[126.18, 127.49]	125.44 (13.07)	[124.59, 126.29]	<0.01
(21) Anemic	0.16 (0.37)	[0.15, 0.18]	0.15 (0.36)	[0.13, 0.17]	0.19 (0.39)	[0.16, 0.21]	0.02
(22) HAZ	-1.39 (1.04)	[-1.43, -1.35]	-1.25 (1.04)	[-1.30, -1.19]	-1.59 (1.01)	[-1.66, -1.52]	<0.01
(23) --Stunted (HAZ<-2)	0.28 (0.45)	[0.26, 0.30]	0.23 (0.42)	[0.21, 0.26]	0.34 (0.47)	[0.31, 0.37]	<0.01
(24) WAZ	-1.39 (1.05)	[-1.43, -1.34]	-1.27 (1.05)	[-1.33, -1.21]	-1.55 (1.02)	[-1.62, -1.48]	<0.01
(25) --Underweight (WAZ<-2)	0.26 (0.44)	[0.25, 0.28]	0.23 (0.42)	[0.21, 0.25]	0.31 (0.46)	[0.28, 0.34]	<0.01
(26) BmiAZ	-0.59 (0.99)	[-0.63, -0.55]	-0.55 (1.01)	[-0.61, -0.50]	-0.64 (0.96)	[-0.7, -0.58]	0.04
(27) --Malnourished (BmiAZ<-2)	0.06 (0.25)	[0.05, 0.07]	0.06 (0.23)	[0.05, 0.07]	0.07 (0.26)	[0.06, 0.09]	0.19
***School absence and performance***							
(28) School absence	0.13 (0.34)	[0.12, 0.14]	0.11 (0.31)	[0.09, 0.13]	0.16 (0.37)	[0.14, 0.18]	<0.01
(29) Standardized math test score	52.64 (22.04)	[51.72, 53.57]	56.4 (21.1)	[55.19, 57.51]	47.49 (22.35)	[55.19, 57.51]	<0.01
(30) --Failure (Score<60)	0.62 (0.49)	[0.60, 0.64]	0.56 (0.50)	[0.53, 0.59]	0.70 (0.46)	[0.53, 0.59]	<0.01

Source: authors’ survey.

Note: “na” stands for not available.

### Cognitive Ability

A total of 63 percent of children had a Working Memory Index (WMI) that was either “extremely low” (<70) or “borderline” (70–79). The breakdown shows that 13 percent of children scored “extremely low” on the WMI portion of the test, and 50 percent of children scored in the “borderline” range.

A total of 36 percent of children had a Processing Speed Index (PSI) that was either “extremely low” (<70) or “borderline” (70–79). The breakdown shows that 10 percent of children scored “extremely low” on the PSI portion of the test, and 26 percent of children scored in the “borderline” range.

### Nutritional Indicators

We find that 16 percent of our sample children are anemic (Table 2), 28 percent are stunted (HAZ < -2), 6 percent are malnourished (BMI-for-age < -2), and 26 percent are underweight (WAZ < -2).

### School Absence and Performance

Around 13 percent of children in our sample had been absent from school at least once during the most recent semester. The average child in our sample earned a failing score on the TIMSS test (score < 60), scoring an average of 52.6 out of 100 on the TIMSS test (Table 2).

### STH Infection and Cognitive Ability

**Fig 2:**
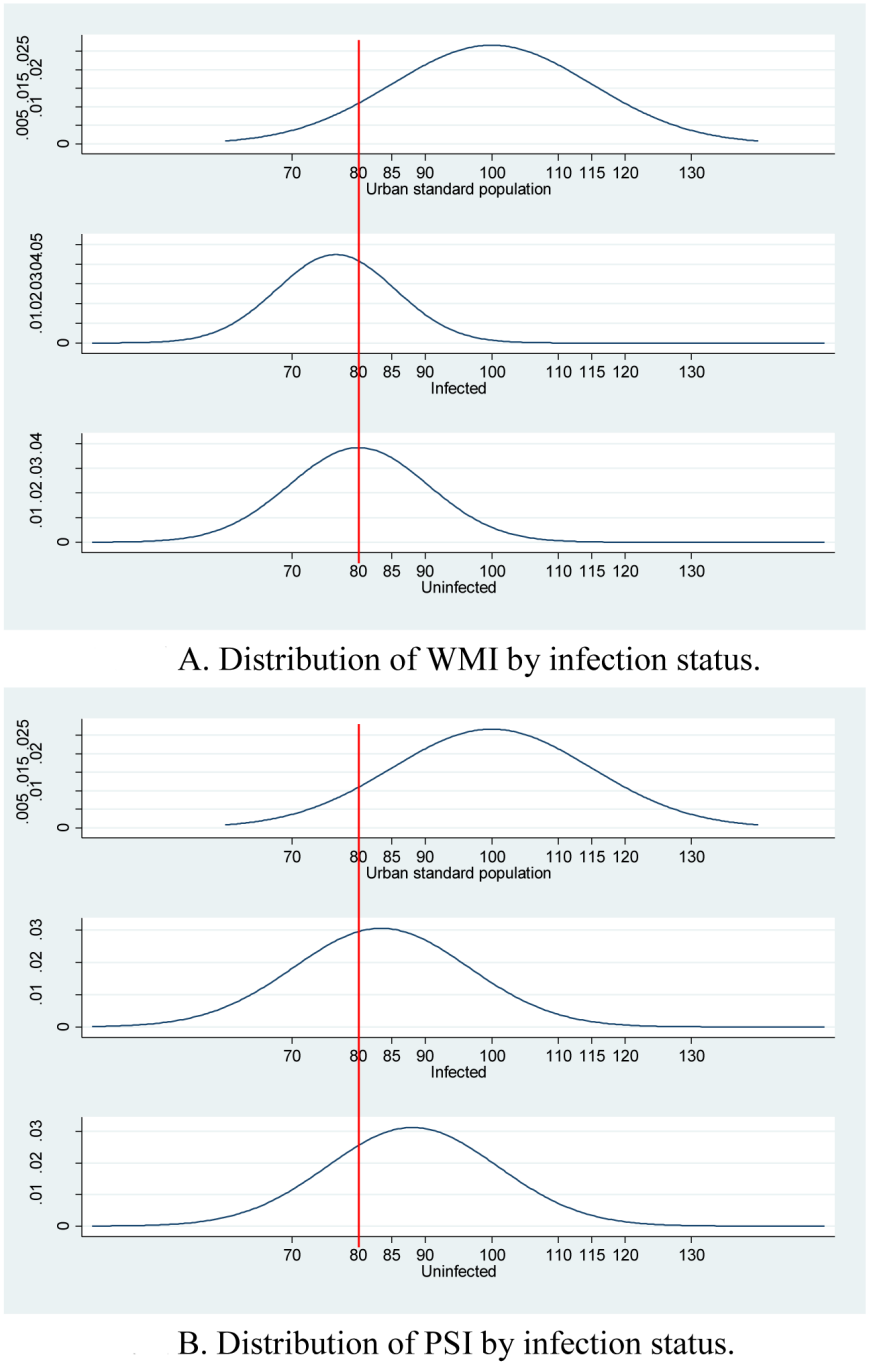
Distribution of cognition by infection status. Note: Any scores to the left of the boarder-line (at 80) mean “delay”.

There are significant differences in children’s cognitive ability between infected children and uninfected children (Fig 2 and Table 2). Our data show that the mean WMI of infected children is 76.6, significantly lower than that of the uninfected group (80.0, p < 0.005). Moreover, 71 percent of infected children had an “extremely low” or “borderline” WMI, significantly higher than that of uninfected children (57 percent, p < 0.005). These differences remain statistically significant after controlling for confounding factors (Table 3).

**Table 3:** Association between STH infection and cognitive ability, nutritional indicators, school absence and performance (infected with any of the 3 types of STHs).

	Coefficient or Odds Ratio (95% CI)^a^ ^,^ ^b^	Effect Size^c^	P-Value^d^
***Reference group*: *no infection with any of the 3 types of STHs***			
***Cognitive ability***			
(1) Working memory index	-2.18 (-3.55, -0.81)	0.011	0.000
(2) Processing speed index	-2.97 (-4.79, -1.14)	0.011	0.000
***Nutritional indicators***			
(3) Hb	-1.33 (-3.23, 0.56)	0.003	0.051
(4) Anemic	1.28 (0.88, 1.87)	1.282	0.067
(5) HAZ	-0.22 (-0.36, -0.08)	0.010	0.000
(6) WAZ	-0.21 (-0.34, -0.07)	0.009	0.000
(7) BmiAZ	-0.09 (-0.23, 0.06)	0.002	0.089
***School absence and performance***			
(8) School absence	1.42 (0.92, 2.18)	1.417	0.027
(9) Standardized math test score	-7.71 (-10.90, -4.53)	0.028	0.000

Notes:

^a^ Estimated with multivariate regressions adjusted for student characteristics (gender, age, boarding status, ethnicity); student eating and sanitation habits (ever eats uncooked meat / vegetables, ever drinks unboiled water); as well as household and family characteristics (household size, number of siblings, pieces of durable assets, parental migrant status, parental education). Standard errors are adjusted for clustering at the township level. Coefficients are reported in cases of continuous outcome variables (namely, WMI, PSI, Hb, HAZ, WAZ, BmiAZ, Standardized math test score) whereas odds ratio reported in cases of binary outcome variables (namely, anemic and school absence).

^b^ Confidence intervals reported here are based on significance level adjusted for multiple hypotheses testing by the Bonferroni method, which adjusted the customary significance level of alpha (i.e., 0.05) downward to 0.006.

^c^ eta^2 are reported in cases of continuous outcome variables (namely, WMI, PSI, Hb, HAZ, WAZ, BmiAZ, Standardized math test score) whereas odds ratio reported in cases of binary outcome variables (namely, anemic and school absence).

^d^ The Bonferroni method adjusted the customary significance level of 0.05 and 0.01 downward to 0.006 and 0.001, respectively.

Source: Authors’ survey.

Our data show that the mean PSI of infected children is 83.4, significantly lower than that of the uninfected group (88.1, p < 0.005). Moreover, 45 percent of infected children in the infected group had an “extremely low” or “borderline” PSI, significantly higher than that of uninfected children (29 percent, p < 0.005). These differences remain statistically significant after controlling for confounding factors (Table 3).

There are no significant differences between children with *Ascaris* only and children with no infection of any of the three types of STHs in terms of WMI and PSI (See Panel A, Table C in S1 Text). The same is true when we compare children with both *Ascaris* and *Trichuris* against children with *Trichuris* only (Panel D2).

However, compared with children with no infection of any of the three types of STHs, children with *Trichuris* only infection had significantly lower WMI (p<0.001) and PSI (p<0.001) after controlling for confounding factors (Panel B). The same strong relationship remains when we compare children with both *Ascaris* and *Trichuris* against children with no infection with any of the three types of STHs (Panel C).

Our data also show that children with a co-infection of both *Ascaris* and *Trichuris* had significantly lower PSI (p<0.001) than children with *Ascaris* only infection, after controlling for confounding factors. However, there is no significant difference in WMI between these two groups of children (Panel D1).

### STH Infection and Nutritional Indicators

There are significant differences in terms of both mean hemoglobin level and mean anemia rate between infected and uninfected children (Table 2). The hemoglobin level among infected children is 126.8 g/L, significantly higher than that among the uninfected group (125.4 g/L, p < 0.01). Meanwhile, the anemia rate among infected children is 19 percent, significantly higher than that among the uninfected group (15 percent, p < 0.05). However, after controlling for confounding factors, the differences between infected children and their uninfected peers were found to be statistically insignificant (Table 3).

There are significant group differences in height-for-age z-scores (HAZ) between infected and uninfected children (Table 2). The mean HAZ among infected children is -1.59, compared with -1.25 among uninfected children (p < 0.005). An average of 34 percent of infected children are stunted, compared with 23 percent of uninfected children (p < 0.005). After controlling for confounding factors, the difference in HAZ remains statistically significant (Table 3, p < 0.001).

There are significant group differences in weight-for-age z-scores (WAZ) between infected and uninfected children (Table 2). The mean WAZ among infected children is -1.55, compared with -1.27 among uninfected children (p < 0.005). An average of 31 percent of infected children are underweight, compared with 23 percent of uninfected children (p < 0.005). After controlling for confounding factors, the difference in WAZ remains statistically significant (Table 3, p < 0.005).

The mean BMI-for-age z-score among infected children is -0.64, compared with -0.55 among uninfected children (p < 0.05). The proportion of being malnourished does not vary significantly between infected and uninfected children (Table 2). Similar to the cases of hemoglobin level and anemia rate, after controlling for confounding factors, the difference in BMI-for-age z-scores becomes statistically insignificant (Table 3).

There are no significant differences between children with *Ascaris* only and children with no infection of any of the three types of STHs in terms of mean hemoglobin level, mean anemia rate, HAZ, WAZ and BmiAZ (Panle A, Appendix Table 3). The same is true when we compare children with both *Ascaris* and *Trichuris* against children with *Trichuris* only (Panel D2).

However, compared with children with no infection of any of the three types of STHs, children with *Trichuris* only infection had significantly lower Hb (p<0.001), lower HAZ (p<0.001) and lower WAZ (p<0.001) after controlling for confounding factors although their mean anemia rate and BmiAZ are not statistically different (Panel B). Our data also show that children with a co-infection of both *Ascaris* and *Trichuris* had significantly lower HAZ (p<0.001) and lower WAZ (p<0.001) than children with no infection of any of the three types of STHs although their mean hemoglobin level, mean anemia rate and BmiAZ are not statistically different (Panel B). Finally, compared with children with *Ascaris* only infection, children co-infected with both *Ascaris* and *Trichuris* also had significantly lower WAZ (p<0.001), but there are no significant differences in terms of mean hemoglobin level, mean anemia rate, HAZ or BmiAZ (Panel D1).

### STH Infection and School Absence and Performance

The absence rate among infected children is 16 percent, compared to 11 percent among uninfected children (p < 0.005) (Table 2). This difference becomes statistically insignificant after controlling for confounding factors (Table 3).

The mean TIMSS score among infected children is 47.5, compared with 56.4 among uninfected children (p < 0.005). A total of 70 percent of infected children failed the TIMSS test (scores < 60), compared with 56 percent of uninfected children (p < 0.005) (Table 2). These differences remain statistically significant after controlling for confounding factors (Table 3, p < 0.001).

Compared to children without infection of any of the three types of STHs, children with *Ascaris* only infection see no difference in the incidence of school absence (See Panel A, Table C in S1 Text). The same is true when we compare children with *Trichuris* only (Panel B) or children with a co-infection with both *Ascaris* and *Trichuris* against children with no infection of any of the three types of STHs (Panel C). Similarly, our data show no significant difference in incidence of school absence between children with a co-infection with both *Ascaris* and *Trichuris* with children with *Ascaris* only (Panel D1) or *Trichuris* only (Panel D2).

Our data also show that there is no difference in standardized test scores between children with *Ascaris* only infection and children with no infection of any of the three types of STHs, after controlling for confounding factors (See Panel A, Table C in S1 Text). The same is true when we compare children with a co-infection of both *Ascaris* and *Trichuris* to children with *Trichuris* only infection (Panel D2).

However, compared with children with no infection of any of the three types of STHs, children with *Trichuris* only infection or children had lower standardized test scores (p<0.001), after controlling for confounding factors (Panel B). The same strong correlation holds when we compare children with a co-infection of both *Ascaris* and *Trichuris* against children with no infection of any of the three types of STHs (Panel C). Our data also show that children with a co-infection of both *Ascaris* and *Trichuris* had lower standardized test scores (p<0.001) than children with *Ascaris* only infection (Panel D1).

## Discussion

In this paper we document the prevalence of STHs using results from stool sampling and socioeconomic testing of 2,179 school children living in seven nationally-designated poverty counties in Qiandongnan prefecture in Guizhou province. We observed that 42 percent of the sample children were infected with one or more of the three types of STH—*Ascaris*, *Trichuris*, and hookworm. This prevalence is consistent with previous, smaller-scale studies in China [2,3], but is more than twice the observed STH prevalence from the National Survey on Current Status of the Important Parasitic Diseases in Human Population in 2004 [25]. According to the WHO treatment guidelines, the prevalence we document warrants mass treatment.

We also document children’s cognitive ability, nutritional indicators, school absence and performance. Our data show that sample children are lagging far behind the international standard in terms of each of these measured outcomes. We further found that after controlling for a set of socioeconomic confounders, infection with one or more STHs is associated with worse cognitive ability (in terms of WMI and PSI), worse nutritional status (in terms of HAZ and WAZ), and worse school performance (in terms of standardized math test scores). Without implying there is any causal link per se, this study also presents evidence that infection with *Trichuris*, either infection with *Trichuris* only or co-infected with *Trichuris* and *Ascaris*, makes children experience worse cognitive, nutritional and schooling outcomes than their uninfected peers or children infected with only *Ascaris*.

These results are consistent with findings from other epidemiological studies and randomized controlled trials examining the relationship between STH infections and cognitive ability [3, 10–14, 26], nutritional indicators [2, 3, 13, 27–32], and school performance [33–34]. In a study of children between the ages of 5 and 14 years in China, Shang reported that STH infection was associated with high incidence of malnourishment, stunting and anemia. The Shang study also found infections were correlated with worse performance on WMI and PSI [3]. These observations support our findings that infections with one or more STHs are associated with worse cognitive ability, worse nutritional status and worse school performance than their uninfected peers. A randomized controlled trial of STH infected children who were treated with albendazole demonstrated less school absenteeism than children in the comparison group who were not treated with albendazole [35]. However, we do not find any strong correlation between STH infection with school absenteeism.

A previous cross-sectional study among schoolchildren in Brazil suggested that polyparasitised children experience worse cognitive outcomes than children with only one helminth infection [14]. We attempted to assess the effects of polyparasitism on child development outcomes, specifically cognitive ability, nutritional indicators, school absence and performance. To do so, a method was developed whereby development outcomes were compared between children with co-infections of STHs and children with only one STH infection. The findings suggest that infection with *Trichuris*, either infection with *Trichuris* only or co-infected with *Trichuris* and *Ascaris*, makes children experience worse cognitive, nutritional and schooling outcomes than their uninfected peers or children infected with only *Ascaris*.

Our study has several limitations. First, due to budgetary constraints, we collected two stool samples per child (on consecutive days), rather than three samples per child. While we believe that two samples adequately allows for the cyclical nature of roundworms’ egg laying patterns, three samples may have allowed for even greater sensitivity in the detection of the true prevalence of infection. Second, while we made every effort to keep samples refrigerated for as long as possible between sample production and laboratory testing, the samples were not produced on site, and therefore children may have waited up to several hours before delivering their samples to the nearest refrigeration facilities (either at the village clinic or at the school). This waiting period was outside of our control, but may have contributed to the degradation of hookworm eggs. Since both of these limitations may have resulted in an underestimate of total STH prevalence, the estimates presented here should be considered to be a lower bound. A third study limitation is that we were unable to collect data on the intensity of infection in the sample areas.

Our study shows that STH infection still poses a significant health challenge among children living in poor, rural, ethnic areas of southwest China. Given the important linkages we find between STH infection and a number of important child health and educational outcomes, we hope that our results will contribute positively to the debate surrounding the recent Cochrane report [1]. Although our results are correlational, we believe that the strength of the correlations is striking, and indicates a need for more rigorous research on the impacts of STH treatment on child outcomes.

Due to the cross-sectional nature of our data, we are unable to identify the precise reasons behind the linkages we observe through this study. However, we can speculate that one possible explanation might be that STH infections lead to nutritional deficits, which in turn contribute to cognitive impairments. Another possible explanation might be that children from disadvantaged households are both more likely to have poor sanitation practices that contribute to chronic STH infection and are also more likely to have poor nutritional intake that may lead to worse cognitive performance.

## Supporting Information

S1 Checklist STROBE checklist. (PDF)

S1 Text Supporting Information tables. Table A. Characteristics and application of the cognitive tests. Table B. Variable definitions. Table C. Association between STH infection and cognitive ability, nutritional indicators, and school performance (by STH type and combination).
(DOC)
