# Supplementary material for: Soil-Transmitted Helminths in Southwestern China: A Cross-Sectional Study of Links to Cognitive Ability, Nutrition, and School Performance among Children
Source: PLoS Negl Trop Dis. 2015 Jun 25;9(6):e0003877. doi: 10.1371/journal.pntd.0003877 (PMC4481344; doi:10.1371/journal.pntd.0003877)
Supplement: S1 Text — Table A. Characteristics and application of the cognitive tests. Table B. Variable definitions. Table C. Association between STH infection and cognitive ability, nutritional indicators, and school performance (by STH type and combination). (DOC) [file pntd.0003877.s002.doc]

Table A. Characteristics and Application of the Cognitive Tests

| **Test** | **Method** |  | **Type** |  | **Skills** |
| --- | --- | --- | --- | --- | --- |
| Digit span | Proctor verbally states numbers; child repeats them back in same order and in inverse order. |  | Verbal test |  | Measures auditory short-term memory, sequencing skills, attention, and concentration |
| Letter Number Sequencing | Proctor verbally states sequences of random letters and digits; child repeats the digits in numerical order then the letters in alphabetical order |  | Verbal test |  | Measures sequencing, mental manipulation, attention, short-term auditory memory |
|  |  |  |  |  |  |
| Coding | Child is shown a legend where numbers of signs are associated with shapes; child is presented with scenarios involving matching according to the legend within a specific time limit (120 seconds) |  | Non-verbal test, performance test |  | Measures processing speed, short-term memory, perceptual abilities, motor coordination, speed |
| Symbol search | Child scans a search group and indicates whether the target symbol(s) matches any of the symbols in the search group within a specific time limit (120 seconds) |  | Non-verbal test, performance test |  | Measures processing speed, short-term visual memory, visual-motor coordination |

Source: WISC-IV, Weschsler Intelligence Scale for Children-Fourth Edition.

Table B. Variable Definitions

| **Variable** | **Description** |
| --- | --- |
| ***STH Infection*** |  |
| Any STH infection | Child is infected with any of the three types of STH: *Ascaris*, hookworm, or *Trichuris* (1=yes, 0=no) |
| Ascaris | Child is infected with *Ascaris* (1=yes, 0=no) |
| Hookworm | Child is infected with hookworm (1=yes, 0=no) |
| Trichuris | Child is infected with *Trichuris* (1=yes, 0=no) |
| ***Cognitive ability*** |  |
| Working memory index | Standardized score on the working memory module of the WISC-IV |
| Processing speed index | Standardized score on the processing speed module of the WISC-IV |
| ***Nutritional indicators*** |  |
| Hb | Hemoglobin concentrations (g/L) |
| Anemic | Hb<115 g/L (1=yes, 0=no) |
| HAZ | Height-for-age z-score |
| WAZ | Weight-for-age z-score |
| BmiAZ | BMI-for-age z-score |
| ***School absence and performance*** |  |
| School absence | Ever been absent from school this semester (1=yes, 0=no) |
| Standardized math test score | % of questions answered correctly on standardized math test |
| ***Correlates*** |  |
| ***-Individual characteristics*** |  |
| Dewormed in past 6 months | Child has taken anti-helminth medicine in past 6 months (1=yes, 0=no) |
| Dewormed in past 12 months | Child has taken anti-helminth medicine in past 12 months (1=yes, 0=no) |
| Female | Child is female (1=yes, 0=no) |
| Age | Age of child, years |
| Boarder | Boarding student this semester (1=yes, 0=no) |
| Minority | Child is non-Han ethnic minority (1=yes, 0=no) |
| --Dong | Child is Dong minority (1=yes, 0=no) |
| --Miao | Child is Miao minority (1=yes, 0=no) |
| --Shui | Child is Shui minority (1=yes, 0=no) |
| --Zhuang | Child is Zhuang minority (1=yes, 0=no) |
| --Other minority | Child is minority other than Dong, Miao, Shui, Zhuang or Yao (1=yes, 0=no) |
| ***Eating and sanitation*** |  |
| Wash hands before eating | Washes hands before eating (1=at least sometimes, 0=no) |
| Wash hands after using toilet | Washes hands after using toilet (1=at least sometimes, 0=no) |
| Never eats uncooked vegetables | Never eats uncooked vegetables (1=never, 0=otherwise) |
| Never eats uncooked meat | Never ate uncooked meat (1=never, 0=otherwise) |
| Never drinks unboiled water | Never ate un-boiled water (1=never, 0=otherwise) |
| Never being outside with bare feet | Child goes outdoors with bare feet in summer (1=never, 0=otherwise) |
| Dirt floor | Household has dirt floor (1=yes, 0=no) |
| Own toilet | Household has own toilet (1=yes, 0=no) |
| Dirt-based latrine | Household toilet is dirt-based latrine (1=yes, 0=no) |
| Use night soil | Household uses night soil in production (1=yes, 0=no) |

| ***Household characteristics*** |  |
| --- | --- |
| Household size | No. of family members (person) |
| Siblings | No. of siblings (person) |
| Pieces of durable assets | Pieces of durable assets owned by the household |
| Neither parent present | Neither parent lives with the child at present (1=neither, 0=otherwise) |
| Mother secondary school | Mother finished secondary school or above  (1=yes, 0=no) |
| Father secondary school | Father finished secondary school or above  (1=yes, 0=no) |

Table C. Association between STH infection and cognitive ability, nutritional indicators, and school performance (by STH type and combination)

|  | Coefficient or Odds Ratio (95% CI)^a,b^ | Effect size^c^ | P-Value^d^ |
| --- | --- | --- | --- |
| ***Panel A. Infected with Ascaris only***  ***Reference group: no infection with any of the 3 types of STHs*** | | | |
| ***Cognitive ability*** |  |  |  |
| (1) Working memory index | -1.49 (-3.28, 0.30) | 0.004 | 0.021 |
| (2) Processing speed index | -1.33 (-3.56, 0.90) | 0.002 | 0.097 |
| ***Nutritional indicators*** |  |  |  |
| (3) Hb | 0.06 (-2.01, 2.12) | 0.000 | 0.939 |
| (4) Anemic | 0.98 (0.64, 1.49) | 0.975 | 0.868 |
| (5) HAZ | -0.16 (-0.34, 0.03) | 0.004 | 0.017 |
| (6) WAZ | -0.10 (-0.28, 0.07) | 0.002 | 0.100 |
| (7) BmiAZ | -0.01 (-0.18, 0.16) | 0.000 | 0.834 |
| ***School absence and performance*** |  |  |  |
| (8) School absence | 1.26 (0.69, 2.30) | 1.260 | 0.292 |
| (9) Standardized math test score | -3.54 (-7.11, 0.04) | 0.005 | 0.007 |
|  |  |  |  |
| ***Panel B. Infected with Trichuris only***  ***Reference group: no infection with any of the 3 types of STHs*** | | | |
| ***Cognitive ability*** |  |  |  |
| (1) Working memory index | -2.98 (-4.92, -1.04) | 0.011 | 0.000 |
| (2) Processing speed index | -4.17 (-6.99, -1.36) | 0.013 | 0.000 |
| ***Nutritional indicators*** |  |  |  |
| (3) Hb | -3.08 (-5.98, -0.18) | 0.008 | 0.004 |
| (4) Anemic | 1.58 (0.94, 2.65) | 1.580 | 0.015 |
| (5) HAZ | -0.25 (-0.44, -0.07) | 0.007 | 0.000 |
| (6) WAZ | -0.24 (-0.43, -0.06) | 0.007 | 0.000 |
| (7) BmiAZ | -0.12 (-0.33, 0.09) | 0.002 | 0.118 |
| ***School absence and performance*** |  |  |  |
| (8) School absence | 1.44 (0.79, 2.62) | 1.436 | 0.098 |
| (9) Standardized math test score | -9.89 (-14.64, -5.14) | 0.000 | 0.000 |
|  |  |  |  |
| ***Panel C. Infected with Ascaris and Trichuris***  ***Reference group: no infection with any of the 3 types of STHs*** | | | |
| ***Cognitive ability*** |  |  |  |
| (1) Working memory index | -2.72 (-4.61, -0.83) | 0 .008 | 0.000 |
| (2) Processing speed index | -5.29 (-7.97, -2.62) | 0.020 | 0.000 |
| ***Nutritional indicators*** |  |  |  |
| (3) Hb | -2.44 (-5.52, 0.64) | 0.005 | 0.028 |
| (4) Anemic | 1.69 (0.94, 3.03) | 1.690 | 0.014 |
| (5) HAZ | -0.35 (-0.62, -0.08) | 0.013 | 0.000 |
| (6) WAZ | -0.39 (-0.65, -0.14) | 0.016 | 0.000 |
| (7) BmiAZ | -0.20 (-0.43, 0.04) | 0.004 | 0.024 |
| ***School absence and performance*** |  |  |  |
| (8) School absence | 1.79 (0.98, 3.26) | 1.792 | 0.008 |
| (9) Standardized math test score | -13.63 (-19.04, -8.22) | 0.047 | 0.000 |
|  |  |  |  |
| ***Panel D1. Infected with Ascaris and Trichuris***  ***Reference group: infection with Ascaris only*** | | | |
| ***Cognitive ability*** |  |  |  |
| (1) Working memory index | -0.66 (-2.75, 1.43) | 0.001 | 0.380 |
| (2) Processing speed index | -3.14 (-5.84, -0.43) | 0.012 | 0.002 |
| ***Nutritional indicators*** |  |  |  |
| (3) Hb | -2.15 (-5.39, 1.08) | 0.006 | 0.064 |
| (4) Anemic | 1.67 (0.87, 3.19) | 1.670 | 0.029 |
| (5) HAZ | -0.13 (-0.41, 0.15) | 0.003 | 0.205 |
| (6) WAZ | -0.26 (-0.51, -0.01) | 0.014 | 0.005 |
| (7) BmiAZ | -0.21 (-0.43, 0.01) | 0.010 | 0.010 |
| ***School absence and performance*** |  |  |  |
| (8) School absence | 1.48 (0.71, 3.10) | 1.480 | 0.145 |
| (9) Standardized math test score | -9.45 (-15.33, -3.58) | 0.038 | 0.000 |
|  |  |  |  |
| ***Panel D2. Infected with Ascaris and Trichuris***  ***Reference group: infection with Trichuris only*** | | | |
| ***Cognitive ability*** |  |  |  |
| (1) Working memory index | 0.09 (-2.24, 2.42) | 0.000 | 0.914 |
| (2) Processing speed index | -0.75 (-4.06, 2.57) | 0.001 | 0.527 |
| ***Nutritional indicators*** |  |  |  |
| (3) Hb | 0.29 (-3.10, 3.69) | 0.000 | 0.807 |
| (4) Anemic | 1.07 (0.63, 1.82) | 1.071 | 0.720 |
| (5) HAZ | -0.03 (-0.31, 0.24) | 0.000 | 0.732 |
| (6) WAZ | -0.10 (-0.39, 0.18) | 0.002 | 0.308 |
| (7) BmiAZ | -0.07 (-0.31, 0.16) | 0.001 | 0.380 |
| ***School absence and performance*** |  |  |  |
| (8) School absence | 1.31 (0.66, 2.60) | 1.310 | 0.279 |
| (9) Standardized math test score | -2.80 (-8.42, 2.83) | 0.004 | 0.164 |
|  |  |  |  |

Notes:

^a^ Estimated with multivariate regressions adjusted for student characteristics (gender, age, boarding status, ethnicity); student eating and sanitation habits (ever eats uncooked meat / vegetables, ever drinks unboiled water); as well as household and family characteristics (household size, number of siblings, pieces of durable assets, parental migrant status, parental education). Standard errors are adjusted for clustering at the township level. Coefficients are reported in cases of continuous outcome variables (namely, WMI, PSI, Hb, HAZ, WAZ, BmiAZ, Standardized math test score) whereas odds ratio reported in cases of binary outcome variables (namely, anemic and school absence).

^b^  Confidence intervals reported here are based on significance level adjusted for multiple hypotheses testing by the Bonferroni method, which adjusted the customary significance level of alpha (i.e., 0.05) downward to 0.006.

^c^ eta^2 are reported in cases of continuous outcome variables (namely, WMI, PSI, Hb, HAZ, WAZ, BmiAZ, Standardized math test score) whereas odds ratio reported in cases of binary outcome variables (namely, anemic and school absence).

^d^ The Bonferroni method adjusted the customary significance level of 0.05 and 0.001 downward to 0.006 and 0.001, respectively.

Source: Authors’ survey.
